# Teledentistry for Pediatric Dental Emergency: Comparison Between Experienced and Novice Users

**DOI:** 10.3390/bioengineering11111054

**Published:** 2024-10-23

**Authors:** Chih-Chieh Huang, Jung-Wei Chen

**Affiliations:** 1 Independent Researcher, Longview, WA 98632, USA; 2 Advanced Education Program in Pediatric Dentistry, Department of Pediatric Dentistry, Loma Linda University School of Dentistry, Loma Linda, CA 92350, USA

**Keywords:** teledentistry, diagnosis, emergency, general dentists, dental students

## Abstract

Background: During the COVID-19 pandemic, teledentistry was often employed for pediatric emergency treatments. Dental students acted as the first health providers using teledentistry under the supervision of faculties in most hospital-based or university-based medical centers during the lockdown period. The aims of this study were to assess the quality of using teledentistry among general dentists (GDs) and dental students (DSs) for managing pediatric dental emergencies. Methods: In total, 60 DSs and 85 GDs were recruited in this study. Each participant was assigned to one of five teledentistry emergency scenarios in pediatric dentistry using a stratified random assignment method. Teledentistry with five emergency scenario simulations was used to evaluate the quality of diagnosis (QD) and treatment (QT) and the detailed information (DI) among all participants. A post-visit survey collected demographic data, usability, confidence in diagnosis (CD), and confidence in treatment recommendation (CT). Descriptive and inferential statistics data were analyzed. The significance level was set as *p* < 0.05. Results: Overall, the study showed that GDs and DSs can use teledentistry to provide good quality of diagnosis (74.5%) and treatment recommendations (77.2%). When encountering pediatric dental emergency scenarios, GDs scored significantly higher (*p* < 0.001) than DSs regarding QD, QT, CD, and CT. Significant differences were noted in QD (*p* < 0.001), QT (*p* < 0.001), CD (*p* = 0.045), and DI (*p* = 0.042) when the subjects encountered five different scenarios. Significant correlations were noted between the amount of detailed information subjects obtained with the quality of diagnosis and treatment recommendation. Confidence in diagnosis is significantly correlated to the quality of diagnosis (*p* = 0.034) and treatment recommendation (*p* = 0.042). However, the confidence in treatment recommendation is not correlated with either QD or QT. Both GDs and DSs hold positive attitudes toward the usability of teledentistry. Conclusions: Teledentistry is effective for diagnosing and managing pediatric dental emergencies. Experienced users provided a better quality of visit compared to novice users, so dental students should be supervised when performing a teledentistry visit.

## 1. Introduction

Teledentistry, according to the American Dental Association’s (ADA) Comprehensive Policy Statement, refers to using a broad variety of technologies and tactics to deliver virtual medical, health, and education services in the dental field. Teledentistry can include patient care and education delivery using different modalities [1,2] and has the ability to improve access to oral health care, improve the delivery of oral health care, and lower its costs [3,4,5]. For decades, teledentistry has been used in different specialties in dentistry [3,5,6,7,8,9,10,11,12]. The advantage of teledentistry is also proven by most studies in that the use of this resource for diagnostic purposes can reduce costs and the travel time to consult a specialist personally [5].

During the COVID-19 pandemic, teledentistry was used a lot due to all non-emergency oral health care being recommended to be postponed, with only emergency care being provided [13]. Dental students acted as the first health providers using teledentistry under the supervision of faculties in many hospital-based or university-based medical centers during the lockdown period. Implementing teledentistry in the pediatric field was even more important because children present a high risk of asymptomatic transmission of COVID-19 due to the prolonged incubation period of the virus. It not only leads children to receive proper management but also ensures the safety of the patient and clinician by sustaining a protective physical barrier.

In today’s technologically advanced landscape, the integration of artificial intelligence (AI) in teledentistry is transforming the delivery of dental care. AI enables remote screening, accurate diagnosis, efficient record-keeping, effective triaging, and continuous patient monitoring through smart devices. This not only allows dental professionals to serve a larger and underserved population but also facilitates a shift from a curative model to a preventive and personalized approach. By leveraging AI technologies, dentists can offer tailored interventions that enhance patient outcomes and promote equitable access to quality oral health care [14].

The value of accurate diagnosis and recommendations via teledentistry is crucial. The quality of diagnosis traditionally was evaluated by sensitivity (true positive/all positive findings) and specificity (true negative/all negative findings). It not only ensures the patient’s safety, but also brings significant merits since diagnostic error has been defined as a major health care issue [15], which causes a substantial source of morbidity, mortality, and costs. Some of the dental schools have dental students undergo teledentistry training with both academic and clinical experience. Currently, great numbers of general dentists also integrate teledentistry into daily practice, especially when patients call for emergency appointments.

The purpose of this study is to evaluate dental students (DSs) and general dentists (GDs) using teledentistry when encountering a pediatric emergency appointment. We will compare the accuracy of diagnosis, recommendations, and confidentiality of using teledentistry with its associated factors, as well as the usability between DSs and GDs. We aim to find out the factors that can elevate the quality of teledentistry provided by DSs and GDs, which will bring benefit to pediatric patients.

## 2. Materials and Methods

This study was reviewed and approved by the Institutional Review Board at Loma Linda University, Loma Linda, CA, USA (IRB #5220048). The sample size was determined by power analysis with a 90% power, an alpha error of 0.05, an effect size of 0.8, and an n equal to 145 using GPower ^TM^ 3.1 software (Universitat Kiel, Kiel, Schleswig-Holstein, Germany). All subjects (third-year and fourth-year DSs and GDs in specific areas) were recruited between August 2022 and September 2022. Participation in this study was voluntary, and subjects were able to choose if they wanted to stop participating at any time.

Prior to the research, five scenario scripts were developed to simulate a pediatric teledentistry setting, which included two trauma scenarios (tooth avulsion and incisal fracture), two pain scenarios (cellulitis and pulpitis), and one control (gingivitis) scenario. The scripts included the photos and conversations between the patient and dentist, which simulated the scenario. The two trauma scripts were written based on the IAPT trauma guideline and AAPD trauma assessment form, which include the history, examination, and assessment. The other three scripts were written based on the reference manual of pediatric dentistry simulating real-life dental visits. The scripts were provided to a standard patient who acted as the legal guardian. The standard patient needed to answer the questions asked by the subject based on the scenarios’ scripts, and non-included questions that were not listed in the scripts were answered with a negative response. All subjects were stratified and randomly assigned to one of five scenarios for teledentistry. The subjects were interviewed at the routine clinical teledentistry setting with the standard patient in another controlled room with the same teledentistry equipment for all encounters. The teledentistry interview occurred via an online one-to-one synchronous video meeting with password protection using Zoom (ver. 5.12.2, San Jose, CA, USA). The subject needed to take part in a real-life E-visit to collect data and make diagnosis and treatment recommendations. After the scenario was completed, all subjects were given an anonymous online usability and confidence survey. The E-visits were recorded and reviewed by one single reviewer. To assess consistency, the single reviewer re-examined a subset of videos that can represent the samples. The videos were randomly selected, and the intra-rater reliability was calculated based on this reassessment, yielding a reliability score above 0.9. Teledentistry index (TDI) scores were collected. All documents were encrypted and saved in a password-protected hard drive (Western Digital My Passport, 5 TB). The documents were stored in a double-locked room.

### 2.1. Teledentistry Index (TDI)

The TDI includes the following: (1) quality of diagnosis (QD), (2) quality of treatment recommendations (QT), (3) confidence in diagnosis (CD), (4) confidence in treatment recommendations (CT), and (5) percentage of detailed information (DI). QD and QT were scored by a single reviewer based on the score rubrics with a 5-point Likert scale (1 = very poor, 2 = poor, 3 = acceptable, 4 = good, 5 = very good). QD was based on the accuracy of diagnosis and the detail of differential diagnosis. QT was based on treatment planning with management and treatment variations. Both QD and QT are evaluated with IADT trauma guidelines and AAPD trauma assessment forms. CD and CT were collected from the subject’s answers to an online survey using a 5-point Likert scale (1 = not confident at all, 2 = slightly confident, 3 = somewhat confident, 4 = fairly confident, 5 = completely confident). DI information was gathered by evaluating the extent to which participants in the study collected relevant data. This process involved calculating the percentage of essential information obtained by each participant during teledentistry. The assessment was based on three main categories: (1) basic data, which included general patient demographics such as age, gender, and legal guardian; (2) patient-specific information, covering aspects like current medications, medical history, dental history, and any allergies; and (3) chief complaint-related data, which focused on the participant’s ability to elicit information directly related to the patient’s primary issue or reason for seeking care. The percentage of data gathering was calculated based on how many of these critical pieces of information the participant asked for and documented in comparison to what was available in the scripted scenario. This provided a comprehensive measure of how thoroughly each participant gathered the necessary data during teledentistry.

### 2.2. Usability and Confidence Survey

The usability and confidence survey (19 items) included the following: (1) demographic background: five multiple choice questions including age, gender, years in practice, and practice setting; (2) usability: ten multiple choice questions using a 5-point Likert scale and two open-ended questions including the frequency of using teledentistry, helpfulness of the tool, ease of use, and the difficulties encountered; and (3) confidence: two multiple choice questions using a 5-point Likert scale regarding the subject’s confidence toward his or her diagnosis and treatment recommendation.

### 2.3. Statistical Analysis

All of the data were entered in Microsoft^®^ Excel^®^ ver.16.69 (Microsoft Corp., Redmond, WA, USA). Descriptive statistical analyses were performed on TDI variables and the user’s usability and confidence survey. Statistical analysis was performed using SPSS^®^ 26.0 (IBM Corp., Chicago, IL, USA). The Pearson X^2^ test, Mann–Whitney U test, and independent-sample t-test were used to determine if significant differences existed between the DSs and GDs. One-way analysis of variance (ANOVA) with LSD (least significant difference) post hoc tests were used to evaluate DI among the five experimental scenarios, and a Kruskal–Wallis test was used to evaluate differences in QD, QT, CD, and CT among the five experimental scenarios. Statistical significance was set at *p* < 0.05.

## 3. Results

### 3.1. Demographics of Study Participants

A total of 145 participants (n = 85 DSs and n = 60 GDs) were recruited. Each participant was assigned to one of five scenarios using a stratified random assignment method, ensuring that each scenario included an equal distribution of 17 dental students and 12 general dentists. Table 1 summarizes the demographics of the participants. There was a significant difference regarding the age, years in practice, and practice setting between the two groups (*p* < 0.001). Of the 145 participants, the majority of GDs in the study had been in practice for more than ten years, primarily positioned as full-time faculty or in a private practice setting. Among 85 students, 5 of them answered that they had work experience before enrolling in school. There is no significant difference observed regarding gender among the two groups. The sensitivities of the diagnosis and treatment recommendation are both higher in GDs than in DSs, which is 83.3% versus 54.4% in diagnosis and 93.8% versus 57.4% in treatment recommendation.

### 3.2. Teledentistry in Different Scenarios

Overall, 74.5% of QD and 77.2% of QT were above the acceptable score. The sensitivities of QD and QT are 68.97% and 71.55%, respectively. The specificities of QD and QT are both 100%. When removing the negative control scenario (gingivitis), 68.1% QD and 71.6% QT were above the acceptable score, which showed that QD and QT were reduced. Significant differences were noted in both QD and QT among the five different scenarios (*p* < 0.001), with the avulsion scenario having the lowest score (Table 2). This means that we need to improve our education in avulsion trauma. QD of cellulitis was second to the lowest, but its QT was second to the highest. This finding indicated that when facing severe pain or obvious life-threatening scenarios, subjects can make appropriate treatment recommendations, although the diagnosis might not be correct.

When comparing the TDI in five different scenarios, a significant difference was noted in QD (*p* < 0.001), QT (*p* < 0.001), CD (*p* = 0.045), and DI (*p* = 0.042) (Table 2). When we removed the control, a significant difference was noted only in QT (*p* < 0.001), CD (*p* = 0.024), and DI (*p* = 0.015). This means that subjects can differentiate between treatment-needed scenarios and normal scenarios by using teledentistry. They can still make appropriate treatment recommendations even if they are not able to make the exact diagnosis when applying it to treatment-needed scenarios.

### 3.3. Comparison Between General Dentists and Dental Students

**Figure 1:**
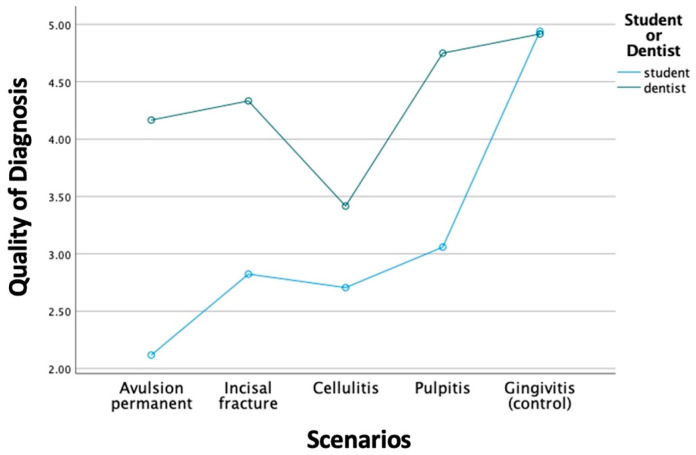
Comparison of QD between GDs and DSs.

**Figure 2:**
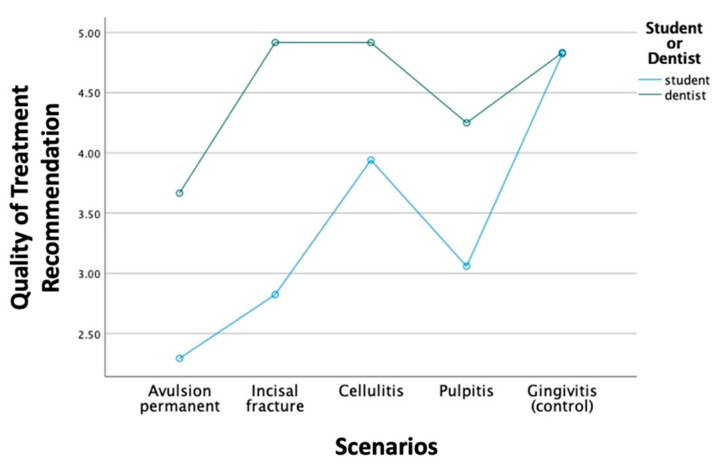
Comparison of QT between GDs and DSs.

When comparing GD and DS groups, a significant difference was observed in both QD and QT (*p* < 0.001). GDs showed significantly better QD and QT than DSs in all the experimental scenarios, except the control scenario (Figure 1 and Figure 2). The sensitivities of QD and QT in the GD group are both higher than in the DS group, which are 83.3% compared to 54.4% in QD, and 93.8% compared to 57.4% in QT. However, the specificities in QD and QT are 100% in both GD and DS groups.

Table 3 shows the mean TDI scores of GDs and DSs among the different scenarios. For the DS group, a significant difference was noted in QD (*p* < 0.001), QT (*p* < 0.001), DI (*p* = 0.033) with the control, and QT (*p* = 0.014), DI (*p* = 0.012), CD (*p* = 0.033) when taking out the control. The difference in QD significance showed that DSs were able to use teledentistry diagnosis normal findings, but they might need their supervisor when facing treatment-needed scenarios. For the GD group, a significant difference in QD (*p* = 0.003), QT (*p* < 0.001) was noted with the control, and QD (*p* = 0.019), QT (*p* < 0.001) and CT (*p* = 0.044) when taking out the control. The cellulitis scenario received the lowest score in QD, whereas the avulsion scenario received the lowest score in QT. The results showed that although the QD of cellulitis is low, GDs can make good QT. However, GDs can diagnose permanent teeth avulsion correctly, but they lack the knowledge to manage it appropriately.

**Figure 3:**
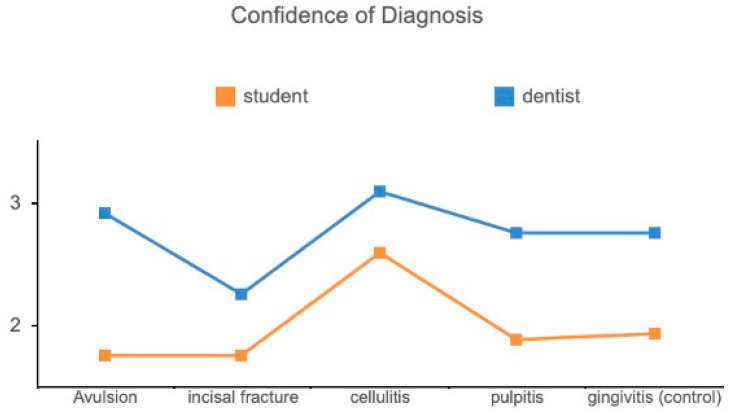
Comparison of CD between GDs and DSs.

**Figure 4:**
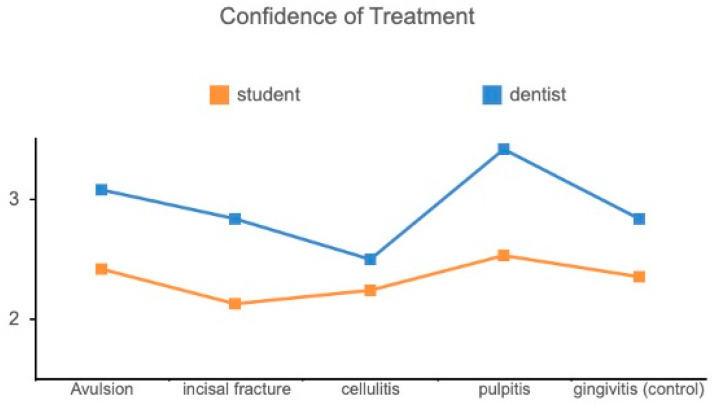
Comparison of CT between GDs and DSs.

There was no significant difference (*p* = 0.144) between DSs and GDs regarding DI. However, an interesting finding was that the correlation became significant between DI and QT (R = 0.206, *p* = 0.027) when taking out the control scenario. This means that when we face disease scenarios, it is important to obtain sufficient information to provide good treatment recommendations. In addition, there was a statistically significant difference between GDs and DSs concerning their confidence in using teledentistry. GDs scored higher in CD (*p* < 0.001) and CT (*p* < 0.001) when using teledentistry in contrast to DSs (Figure 3 and Figure 4).

For the correlation, the subject’s CD and CT, as well as QD and QT, were significantly related to both age and years in practice (Table 4). Among all experimental scenarios, CD was related to QD (*p* = 0.034) and QT (*p* = 0.042), while CT was not related to either QD (*p* = 0.077) or QT (*p* = 0.155). These findings showed that when subjects are confident with their diagnosis, they tend to provide better QD and QT. The confidence in their treatment recommendations does not necessarily provide better diagnosis or treatment options. However, if we only investigate the GD group, the only correlation is years in practice and CD (*R* = 0.364, *p* = 0.004). The longer they practiced, the less confidence was noted, but the scores did not show the same significant increase in their QD or QT. From this, we can infer that when dentists recently graduate, they tend to be more confident since it is the transitional stage from students to dentists. As time passes, they tend to become humbler because of their experiences and the more difficult issues that they encounter. For the DS group, QD was related to QT (*R* = 0.583, *p* < 0.001), and CT was related to the DI (*R* = 0.277, *p* = 0.010), which means that when DSs are using teledentistry to interview a patient, asking for more information can help them present their recommendations confidently.

Regarding usability, no statistically significant difference (*p* = 0.085) was noted between DSs and GDs regarding the frequency of using technology/digital products. Statistically significant differences were noted between DSs and GDs in terms of using teledentistry/teleconsultation for patients (*p* = 0.004). Most DSs (73%) indicated that they never use it.

Both GDs and DSs held a positive attitude toward the statement about the usability of teledentistry (Table 5). Regarding the difficulty in establishing the diagnosis and treatment recommendations, there was a significant difference (*p* = 0.002) showing that 43.5% of the students did not ask for photos. This may have led to the significant result that more GDs agreed with the statement that images help in making their diagnosis (*p* = 0.03) and treatment recommendations (*p* = 0.021). Moreover, “lack of X ray”, “image quality and quantity”, and “lack of clinical exam” were the three top difficulties in both groups.

## 4. Discussion

During the pandemic, teleconsultation, telediagnosis, teletriage, and telemonitoring are all sub-units of teledentistry that provide important functions relevant to dental practice [16]. The American Academy of Pediatric Dentistry (AAPD) recommended using teledentistry as a pre-visit screening tool during the COVID-19 pandemic as interim care pathways for managing pediatric dental patients. These pediatric patients and those with special needs may experience pain and dental trauma differing in nature and intensity from their adult counterparts. To prevent the extra visits of children to dental clinics or hospital-based programs, teledentistry plays an important role. Implementation of teledentistry in a private pediatric practice during the COVID-19 pandemic showed that nearly half of the patient’s urgent dental needs could be managed successfully with teledentistry and without an in-person visit [17]. However, there is still a shortage of information to support the quality of using teledentistry as well as the accuracy in diagnosis and treatment planning.

Telemedicine has been implemented in the healthcare system for decades. A study investigated the use of teletriage in pediatric emergency rooms, which showed good sensitivity (82.85%) and high specificity (96.15%) [18]. Our study’s findings are similar to a study by Gurgel-Juarez et al. and a systemic review in 2018 showing that sensitivity and specificity for dental referrals and diagnostic treatment planning were higher than other index/reference tests [19,20]. This study showed that teledentistry can provide good diagnosis (74.5%) and treatment planning (77.2%) (Table 2) with 54.4–93.8% sensitivity and 100% specificity. As a result, using teledentistry to diagnose patients and create treatment plans is comparable to telemedicine, telehealth, and traditional methods.

The primary purpose of this study was to compare DSs with GDs using teledentistry.

At Loma Linda University, teledentistry is integrated into the academic curriculum for third- and fourth-year dental students. Consequently, this study selected third- and fourth-year dental students as its subjects to align with the institution’s educational approach. Overall, this study showed that GDs performed better than DSs, which is also related to age and years in practice. GDs showed higher sensitivity when compared to DSs, with values of 83.3–93.8% and 54.4–57.4%, respectively. The findings are similar to multiple studies in the literature that compared experienced users and novice users [20,21,22,23,24]. Experience could affect the validity of teledentistry. Studies showed that general practicing dentists were familiar with the protocols for treating patients during the COVID-19 outbreak [25] and were observed to have a high awareness of teledentistry as compared to postgraduate and undergraduate dental students [13]. The reason why the CD of the GD group was negatively correlated to years in practice may be that the longer dentists practice, the humbler they tend to be. We suggest that although students can differentiate between normal and treatment-needed situations, they still need supervisors when using teledentistry to diagnose treatment-needed scenarios. Additionally, gathering more DI while using teledentistry can help novice users provide their treatment recommendations more confidently.

Furthermore, with the advancement of technology, novice users can incorporate artificial intelligence into their educational experiences. One study highlights students’ adaptability to technological innovations and emphasizes their potential to improve educational outcomes. Educational institutions should consider promoting critical engagement with and the responsible use of AI in their curriculum design [26].

In our study, there were five scenarios, including trauma and pain. This is based on the fact that oral dental trauma as well as pain due to caries have been proven to be the two most common scenarios happening in pediatric dental emergencies [27,28]. Our study showed that avulsion scenarios scored the lowest in QD and QT (*p* < 0.001), which is similar to a study that stated only 37.1% of general practitioners recommend replanting the avulsed tooth immediately with most of their years in practice being less than 5 years [21]. Another study also reported that undergraduate students lack confidence in managing dental trauma [22]. With the revised trauma guidelines in 2020 by the International Association for Dental Traumatology (IADT) [29], this study adds to the body of evidence that it is important for both experienced and novice dentists to update their knowledge of dental trauma. Another interesting finding was that the cellulitis scenario scored low in QD but high in QT (*p* < 0.001), which means that although subjects are not able to make the exact diagnosis via teledentistry when facing a life-threatening pain scenario, they can provide appropriate recommendations, which involves searching for immediate medical help. A good specificity of teledentistry can be noted in our study’s consistency with multiple studies in the literature, which have proven the advantage of teledentistry in differentiating between non-treatment-needed and treatment-needed scenarios and eliminating unnecessary in-person visits.

A significant correlation between DI and QT in treatment-needed scenarios in this study pointed out the importance of gathering data during teledentistry. The other benefit of it was also mentioned previously, in that asking for DI in teledentistry can help DSs provide their treatment recommendations more confidently. These findings are consistent with a study in 2018 that showed that a lack of information could decrease the dental professional’s confidence in making a diagnosis using a teledentistry system only [20].

The questionnaire of this study showed that both GDs and DSs hold positive attitudes toward the usability of teledentistry. The advantage of teledentistry was also proven by a study in 2021 which showed that by implementing a telephone consultation as the first point of contact, the number of face-to-face appointments required is reduced by over one-third [6]. However, the findings of our study showed that usability does not necessarily relate to the quality of care. Regarding the main difficulties of using teledentistry in our study, the findings were similar to previous studies in the literature that described three of the top obstacles for the subjects: lack of X-rays, image quality and quantity, and clinical exam. This study also highlights the importance of images in teledentistry, which has also been proven to be effective and advocated for by Estai et al. whose study used images in teledentistry to prioritize high-risk children and provide them with a quick treatment pathway and avoid unnecessary referrals or travel [7]. A systemic review in 2022 also pointed out that asynchronous communication and the adoption of smartphones for image capturing are feasible and convenient for the implementation of teledentistry [19].

Despite the numerous strengths of this study, there are some limitations to consider. First, the student subjects were all from the same institution. Certainly, the result will need to be compared with different dental schools. Second, although the subjects were tested under controlled conditions, in real life, an emergency would present certain stressors that could have an impact on the decision-making process. Third, the teledentistry videos were reviewed by a single reviewer in our study, and although intra-rater reliability was achieved, it would be interesting to see the difference in results when scoring using multiple reviewers. Another limitation is that our scenarios were specified for pediatric trauma and pain, with each subject tested for one scenario, which is aimed at preventing the subject from experiencing fatigue. Future studies could apply a wider variety of different scenarios to investigate the same groups of subjects. Our study does not investigate legal, educational, and insurance issues of teledentistry, which are some barriers that still exist for teledentistry currently [2,30,31,32]. Due to the fact that teledentistry is becoming an emerging trend, most states are likely to gradually reform and keep modifying their policies in teledentistry [32]. In addition, future research on the design and development of teledentistry guidelines may help improve its efficiency and overall utility for dentists.

## 5. Conclusions

In conclusion, teledentistry is effective for diagnosing and managing most pediatric dental emergencies, especially with high specificity. When comparing experienced and novice users, the former provided a better quality of visit compared to the latter; so, we suggest that dental students should be supervised when performing a teledentistry visit in dental school. Additionally, novice users can increase their confidence in treatment recommendations by asking for more detailed information during teledentistry visits. This study also highlights the need for improved education in more severe emergency scenarios in dental school curricula, such as avulsion and cellulitis.

## Figures and Tables

**Table 1:** Demographics and usability of subjects.

Characteristic	Dental Students (N = 85)	General Dentists (N = 60)	*p*-Value (X^2^ Test)
**Age**			<0.001 *
20–30 years old	76 (89.4%)	6 (10.0%)	
31–40 years old	9 (10.6%)	25 (41.7%)	
>40 years old	0	29 (48.3%)	
**Years in practice**			<0.001 *
Students	80 (94%)	0	
<2 years	2 (2.5%)	7 (11.6%)	
2–5 years	3 (3.5%)	10 (16.7%)	
6–10 years	0	9 (15%)	
>10 years	0	34 (56.7%)	
**Gender**			**0.365** (**NS**)
Male	43 (50.6%)	26 (43.3%)	
Female	42 (49.4%)	33 (55%)	
Do not want to disclose	0	1 (1.7%)	
**Practice setting**			
Student	85 (100%)	0	<0.001 *
Full-time faculty	0	29 (48.3%)	
Private practitioner	0	29 (48.3%)	
Community health	0	2 (3.4%)	
**Sensitivity of diagnosis**	54.4%	83.3%	
**Sensitivity of treatment planning**	57.4%	93.8%	

N = subject number; * = statistically significant (*p* < 0.05); NS = no significant difference. *p*-value is determined using Pearson chi-square test.

**Table 2:** Descriptive summary of TDI scores of different scenarios (all subjects).

TDI	Avulsion	Incisal Fracture	Cellulitis	Pulpitis	Gingivitis (Control)	*p*-Value
QD	2.97 (±1.64)	3.45 (±1.35)	3.00 (±1.13)	3.76 (±1.50)	4.93 (±0.26)	<0.001 *
QT	2.86 (±1.38)	3.69 (±1.56)	4.34 (±1.26)	3.55 (±1.27)	4.83 (±0.47)	<0.001 *
CD	2.24 (±1.18)	1.97 (±1.12)	2.79 (±0.86)	2.24 (±1.12)	2.28 (±1.07)	0.045 *
CT	2.69 (±1.00)	2.41 (±1.12)	2.34 (±0.90)	2.90 (±1.05)	2.55 (±1.12)	0.150
DI (%)	39.90 (±15.08)	34.14 (±11.50)	39.28 (±9.76)	44.65 (±11.48)	40.17 (±14.48)	0.042 *

TDI = teledentistry index; QD = quality of diagnosis; QT = quality of treatment recommendation; CD = confidence in diagnosis; CT = confidence in treatment recommendation; DI = detailed information; * = statistically significant (*p* < 0.05).

**Table 3:** Mean TDI scores of different scenarios (DSs and GDs).

	Avulsion		Incisal Fracture		Cellulitis		Pulpitis		Gingivitis (Control)	
	DS	GD	DS	GD	DS	GD	DS	GD	DS	GD
QD	2.12 (±1.27)	4.17 (±1.34)	2.82 (±1.13)	4.33 (±1.15)	2.71 (±1.16)	3.42 (±1.00)	3.06 (±1.48)	4.75 (±0.87)	4.94 (±0.24)	4.92 (±0.29)
QT	2.30 (±1.26)	3.67 (±1.15)	2.82 (±1.51)	4.92 (±0.29)	3.94 (±1.52)	4.92 (±0.29)	3.06 (±1.34)	4.25 (±0.75)	4.82 (±0.53)	4.83 (±0.39)
CD	1.76 (±1.15)	2.92 (±0.90)	1.76 (±1.25)	2.25 (±0.87)	2.59 (±0.87)	3.09 (±0.79)	1.88 (±0.86)	2.75 (±1.29)	1.94 (±1.20)	2.75 (±0.62)
CT	2.41 (±1.00)	3.08 (±0.90)	2.12 (±1.05)	2.83 (±1.11)	2.24 (±1.03)	2.50 (±0.67)	2.53 (±1.18)	3.42 (±0.51)	2.35 (±1.17)	2.83 (±1.03)
DI (%)	38.93 (±12.74)	41.27 (±18.42)	30.88 (±11.07)	38.75 (±10.90)	39.64 (±7.19)	38.77 (±12.91)	43.34 (±11.84)	46.49 (±11.18)	38.53 (±12.60)	42.50 (±17.12)

TDI = teledentistry index; DS = dental student; GD = general dentist; QD = quality of diagnosis; QT = quality of treatment recommendation; CD = confidence in diagnosis; CT = confidence in treatment recommendation; DI = detailed information.

**Table 4:** Correlation between TDI and age/years in practice (all subjects).

TDI	Age		Years in Practice	
	Correlation Coefficient	Sig. (2-Tailed)	Correlation Coefficient	Sig. (2-Tailed)
QD	0.309	<0.001 *	0.408	<0.001 *
QT	0.266	0.001 *	0.325	<0.001 *
CD	0.287	<0.001 *	0.278	<0.001 *
CT	0.211	0.011 *	0.262	0.001 *

TDI = teledentistry index; QD = quality of diagnosis; QT = quality of treatment recommendation; CD = confidence in diagnosis; CT = confidence in treatment recommendation; * = statistically significant (*p* < 0.05).

**Table 5:** Usability of subjects.

Usability	Dental Students (N = 85)	General Dentists (N = 60)	*p*-Value (X^2^ Test)
**The teledentistry is helpful in making a diagnosis.**			**0.211**
Strongly agree	7 (8.2%)	5 (8.3%)	
Agree	39 (45.9%)	22 (36.7%)	
Neutral	22 (25.9%)	20 (33.3%)	
Disagree	17 (20%)	10 (16.7%)	
Strongly disagree	0	3	
**The teledentistry is helpful in making a treatment recommendation.**			**0.327**
Strongly agree	7 (8.2%)	5 (8.3%)	
Agree	49 (57.6%)	35 (58.3%)	
Neutral	20 (23.5%)	12 (20%)	
Disagree	9 (10.6%)	5 (8.3%)	
Strongly disagree	0	3 (5%)	
**The teledentistry is easy to use.**			**0.742**
Strongly agree	13 (15.3%)	8 (13.3%)	
Agree	51 (60%)	40 (66.7%)	
Neutral	15 (17.6%)	10 (16.7%)	
Disagree	6 (7.1%)	2 (3.33%)	
Strongly disagree	0	0	
**I like to use teledentistry in my practice.**			**0.059**
I will always use it.	1 (1.2%)	2 (3.33%)	
I will often use it.	22 (25.9%)	7 (11.7%)	
I will sometimes use it.	51 (60%)	34 (56.7%)	
I will rarely use it.	8 (9.4%)	14 (23.3%)	
I will never use it.	3 (3.5%)	3 (5%)	
**Teledentistry will improve the care I provided.**			**0.348**
Strongly agree	8 (9.4%)	4 (6.7%)	
Agree	55 (64.7%)	32 (53.3%)	
Neutral	17 (20%)	19 (31.7%)	
Disagree	4 (4.7%)	5 (8.3%)	
Strongly disagree	1 (1.2%)	0	

N = subject number; *p*-value is determined using Pearson chi-square test.

## Data Availability

Data are unavailable due to privacy or ethical restrictions.
